# Supplementary material for: The estrogen effect; clinical and histopathological evidence of dichotomous influences in dogs with spontaneous mammary carcinomas
Source: PLoS One. 2019 Oct 25;14(10):e0224504. doi: 10.1371/journal.pone.0224504 (PMC6814212; doi:10.1371/journal.pone.0224504)
Supplement: S1 Table — Prognostic variables in 159 dogs with mammary carcinomas. Univariate and multivariable Cox regression analysis. Variables: tumor size (<3,3–5,>5), Tumor grade (1, 2, 3) Histological subtypes: 1: carcinomas, 2: complex carcinomas, 3: carcinoma arising in benign mixed tumor, 4: other types including solid carcinomas, comedocarcinoma, carcinoma and malignant myoepithelioma, anaplastic carcinoma, carcinosarcoma, Who stages (1–4), Presence/absence of vascular invasion, and surgical margins: clean vs incomplete. Endpoint: time to primary metastasis. Hormonal factors (serum estrogen, tumor estrogen receptor and spay status not included. This dataset consist of pooled data from previously published studies (28, 29). *Hazard Ratio. (DOCX) [file pone.0224504.s001.docx]

**Supplemental Table 1 (ST1): Clinical and Histological Prognostic variables for all cases.**

| **Variable** | **Univariate**  **HR^*^ 95 % CI** | | **P-value** | **Multivariable**  **HR^*^ 95 % CI** | | **P-value** |
| --- | --- | --- | --- | --- | --- | --- |
|  |  |  |  |  |  |  |
| **Tumor size <3, 3-5 >5** | Ref category  1.77  5.45 | 0.59-5.26 2.21-13.41 | 0.307 <0.001 | Ref category 1.01  2.44 | 0.13- 8.02 0.40- 14.90 | 0.990  0.330 |
| **Tumor grade -1 -2 -3** | Ref category 2.42  12.97 | 0.98-5.96 5.72-29.40 | 0.054  <0.001 | Ref category 1.92  5.81 | 0.74- 5.00 1.94- 17.37 | 0.180  0.002 |
| **Histological subtype -1**  **-2**  **-3**  **-4** | Ref category 0.14  0.15  3.66 | 0.02- 1.01 0.02- 1.13 1.80- 7.44 | 0.051  0.066  <0.001 | Ref category 0.13  0.19  0.96 | 0.02- 1.04 0.02- 1.48 0.38- 2.45 | 0.054  0.113  0.935 |
| **Who stage -1**  **-2**  **-3**  **-4** | Ref category 2.40  5.00  6.16 | 0.78 -7.33  1.78 -14.04 2.10-18.03 | 0.126  0.002  0.001 | Ref category 2.26  1.52  1.62 | 0.28-18.38 0.21-11.16 0.24-10.92 | 0.445  0.679  0.622 |
| **Vascular invasion -No**  **-Yes** | Ref category 6.53 | 2.70-15.82 | <0.001 | Ref category 1.29 | 0.43-3.85 | 0.648 |
| **Surgical margins -incomplete -clean** | Ref category 0.23 | 0.10-0.56 | 0.001 | Ref category 0.14 | 0.05-0.38 | <0.001 |

*Hazard Ratio

Prognostic variables in 159 dogs with mammary carcinomas. Univariate and multivariable Cox regression analysis. Variables: tumor size (<3,3-5,>5), Tumor grade (1, 2, 3) Histological subtypes: 1: carcinomas, 2: complex carcinomas, 3: carcinoma arising in benign mixed tumor, 4: other types including solid carcinomas, comedocarcinoma, carcinoma and malignant myoepithelioma, anaplastic carcinoma, carcinosarcoma, Who stages (1-4), Presence/absence of vascular invasion, and surgical margins: clean vs incomplete. Endpoint: time to primary metastasis. Hormonal factors (serum estrogen, tumor estrogen receptor and spay status not included. This dataset consists of pooled data from previously published studies (28, 29).
